# Influence of pressure on structure, stability and ionic conductivity of lithium argyrodite solid electrolytes

**DOI:** 10.1038/s44456-026-00009-1

**Published:** 2026-07-22

**Authors:** Jemma L. Cox, Jack M. Hemingway, James A. Quirk, Erli Lu, James A. Dawson

**Affiliations:** 1 Chemistry – School of Natural and Environmental Sciences, Newcastle University, Newcastle upon Tyne, UK; 2 School of Chemistry, University of Birmingham, Birmingham, UK

**Keywords:** Chemistry, Materials science, Physics

## Abstract

Whilst the importance of external pressure in the manufacturing and operation of practical solid-state batteries has been clearly recognised, its impact on the fundamental properties of solid electrolytes remains underexplored. Here, we combine density functional theory and molecular dynamics simulations to investigate the effect of pressure (0–10 GPa) on the structural, electronic and ion transport properties of lithium argyrodite solid electrolytes, Li_6_PS_5_X (X = Cl and/or Br), with different levels of halide mixing and anion site (S^2−^/X^−^) disorder. We show that increased pressure induces systematic decreases in lattice volume and thermodynamic stability for both ordered and disordered systems. Anion disorder narrows the bandgap, which can change dramatically as a function of pressure. The Li-ion conductivities ( ~ 0.54–1.05 S cm^−1^ at 600 K) and activation energies ( < 0.16 eV), remain high and low, respectively, up to ~2 GPa, with particularly pressure-resilient conduction networks observed in the mixed halide and disordered structures. At higher pressures ( > 2 GPa), most compositions show a clear increase in activation energy (0.15–0.42 eV) accompanied by reduced conductivity ( ~ 0.12–0.59 S cm^−1^ at 600 K). These findings establish external pressure, site disorder and halide mixing as interdependent parameters that can be used to tune argyrodite solid electrolytes for high-performance solid-state batteries.

## Introduction

A key component of the transition to sustainable energy solutions is the development of safer and more efficient energy storage technologies^1–5^. One particularly promising advancement is the replacement of conventional organic liquid electrolytes in Li-ion batteries with solid electrolytes, resulting in the creation of solid-state batteries. These next-generation batteries could address the safety concerns associated with flammable liquid electrolytes and offer improvements in performance and a wider range of operating conditions^6–11^. Nevertheless, achieving high ionic conductivity at both the material and device scales remains a significant challenge for the widespread application of solid-state batteries. To date, only a limited number of solid electrolytes have demonstrated the superionic conductivity ( ~ 10 mS cm^−1^) required at room temperature for practical devices, most of which are sulfide based^12–15^.

With Li-ion conductivities comparable to those of conventional liquid electrolytes, lithium argyrodites with the general formula Li_6_PS_5_X (X = Cl and/or Br) have gained considerable attention as promising solid electrolytes for solid-state batteries^16–21^. In addition to their highly promising Li-ion conductivity, Li_6_PS_5_X argyrodites are also notable for their degree of mechanical softness, which facilitates reduced interfacial resistance and improves processability^22,23^. To further enhance the Li-ion conductivity of argyrodite solid electrolytes, research has focused on controlling the degree of S^2−^/X^−^ site disorder, which has been shown to directly influence Li-ion diffusivity and, consequently, the material’s ionic conductivity^24–28^. Both experimental and computational studies indicate that increasing this site disorder lowers the activation energy barrier for Li-ion migration by altering the local anionic charge distribution and introducing charge inhomogeneity^24,29–31^. As a result, the Li-ion jump distances between adjacent cages decrease, leading to a more diffuse Li-ion distribution throughout the three-dimensional lattice, ultimately improving the Li-ion conductivity^28,29,32^.

Additional studies have explored the impact of halide (Cl/Br) mixing on Li-ion transport in Li_6_PS_5_X^33–36^. These studies have revealed substantial enhancements in Li-ion conductivity compared to single-halide (Cl- or Br-only) argyrodites. For example, Kraft et al. reported that Li_6_PS_5_Cl_0.5_Br_0.5_ presented an improved Li-ion conductivity due to its softer lattice^34^. However, the softer bonds resulting from increased halide polarisability were also found to decrease the prefactor of the moving ion, thereby resulting in the requirement for a delicate balance to achieve optimised Li-ion conductivity in these materials. The existence of these often intertwined mechanisms has led to significant debate over the factors that dominate Li-ion conductivity in argyrodite solid electrolytes. Furthermore, the vast number of possible atomic configurations arising from the complex distribution of Li cations and S, Cl, and Br anions within their respective sublattices result in an increase in configurational entropy, which has been proposed as another cause of high Li-ion diffusivity^37–40^.

The application of external pressure is another important consideration for solid electrolyte and solid-state battery design and optimisation, with ~20–100 MPa in external stack pressure typically required to maintain solid-solid contacts between the components^41^. As a key thermodynamic parameter, pressure can modify the crystal lattice, defect concentrations and phase stability, thereby altering ion dynamics. These structural changes, in turn, impact energy barriers for ion migration and the overall ionic conductivity^42–44^. However, despite the fact the real-world applications expose solid electrolytes and solid-state batteries to significant pressures and mechanical stresses during both assembly and operation^44^, their critical properties, including structure, thermodynamic stability and ionic conductivity, are often only assessed under ambient pressure conditions. Two important exceptions to this are the works of Faka et al., who explored the pressure dependence of Li-ion conductivity^44^ and pressure-induced dislocations^43^ in Li_6_PS_5_Br using a range of experimental techniques. Nevertheless, our understanding of pressure effects in argyrodite solid electrolytes (and solid electrolytes more broadly) remains limited, particularly at the atomistic scale.

To rectify this omission, in this study, we investigate the impact of a range of external pressures (0–10 GPa) on the structural, electronic and ion transport properties of Li_6_PS_5_X (X = Cl and/or Br) with varying degrees of halide mixing and S^2−^/X^−^ site disorder. Using density functional theory (DFT) and *ab initio* molecular dynamics (AIMD), we show that increasing pressure leads to systematic lattice compression, reduced thermodynamic stability, bandgap narrowing, lower Li-ion conductivities and higher activation energies. Nevertheless, anion disorder and Br substitution can be used to enhance lattice flexibility and preserve reasonable ionic conductivity under pressures of < 10 GPa, identifying them as key design parameters for pressure-resilient argyrodite solid electrolytes. By developing a comprehensive mechanistic understanding of these interlinking factors, this work helps to guide the design of next-generation argyrodite solid electrolytes with enhanced performance and reliability across a diverse range of operating conditions.

## Results

### Local Structure and Stability

**Fig. 1:**
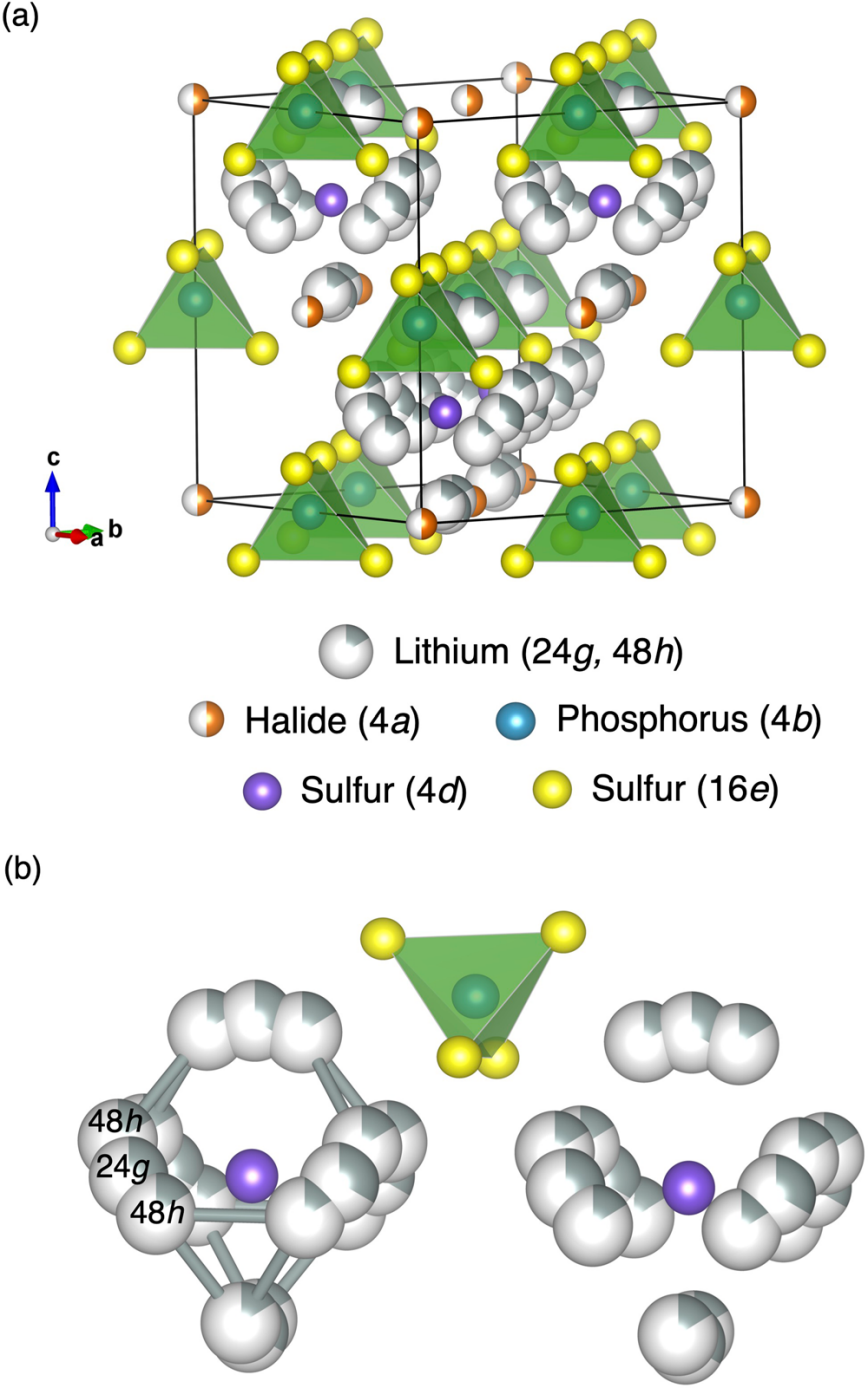
Crystal structure of Li_6_PS_5_X (X = Cl or Br). **a** Fully ordered unit cell of Li_6_PS_5_X. **b** Crystallographic description of the Li-ion cages around the centrally positioned S^2−^.

The crystal structure of Li_6_PS_5_X (X = Cl and/or Br) adopts a face-centred cubic lattice, belonging to the *F*4̅3*m* space group^26^. Fig. 1a illustrates the fully ordered unit cell of Li_6_PS_5_X, in which halide ions are located at the Wyckoff 4*a* positions, while S^2−^ anions occupy the Wyckoff 4 *d* positions. As discussed above, S^2−^ and X^−^ anions can exhibit site disorder by exchanging positions and occupying both 4*a* and 4 *d* sites. The unit cell of Li_6_PS_5_X contains four distinct Li^+^ ‘cages’, with Li ions distributed across 48 *h* and 24 *g* sites, each centred around an S^2−^ anion at the 4 *d* site, as shown in Fig. 1b^26,32^.

To capture the role of anionic disorder, we compare fully ordered configurations (0% S^2−^/X^−^ site exchanged) with partially disordered ones in which three of the four Li^+^ cages per unit cell have their central S^2−^ exchanged with X^−^ (75% S^2−^/X^−^ site exchanged). Previous experimental studies and simulations have shown that such a degree of anion disorder directly influences Li-ion mobility, by expanding Li^+^ cages, reducing inter-cage jump distances, and enhances conductivity by creating more connected diffusion pathways^24,32^. We have therefore focused on these two configurations, fully ordered (0%) and partially disordered (75%), as structural limits for assessing how pressure influences the fundamental properties of argyrodites as solid electrolytes.

**Fig. 2:**
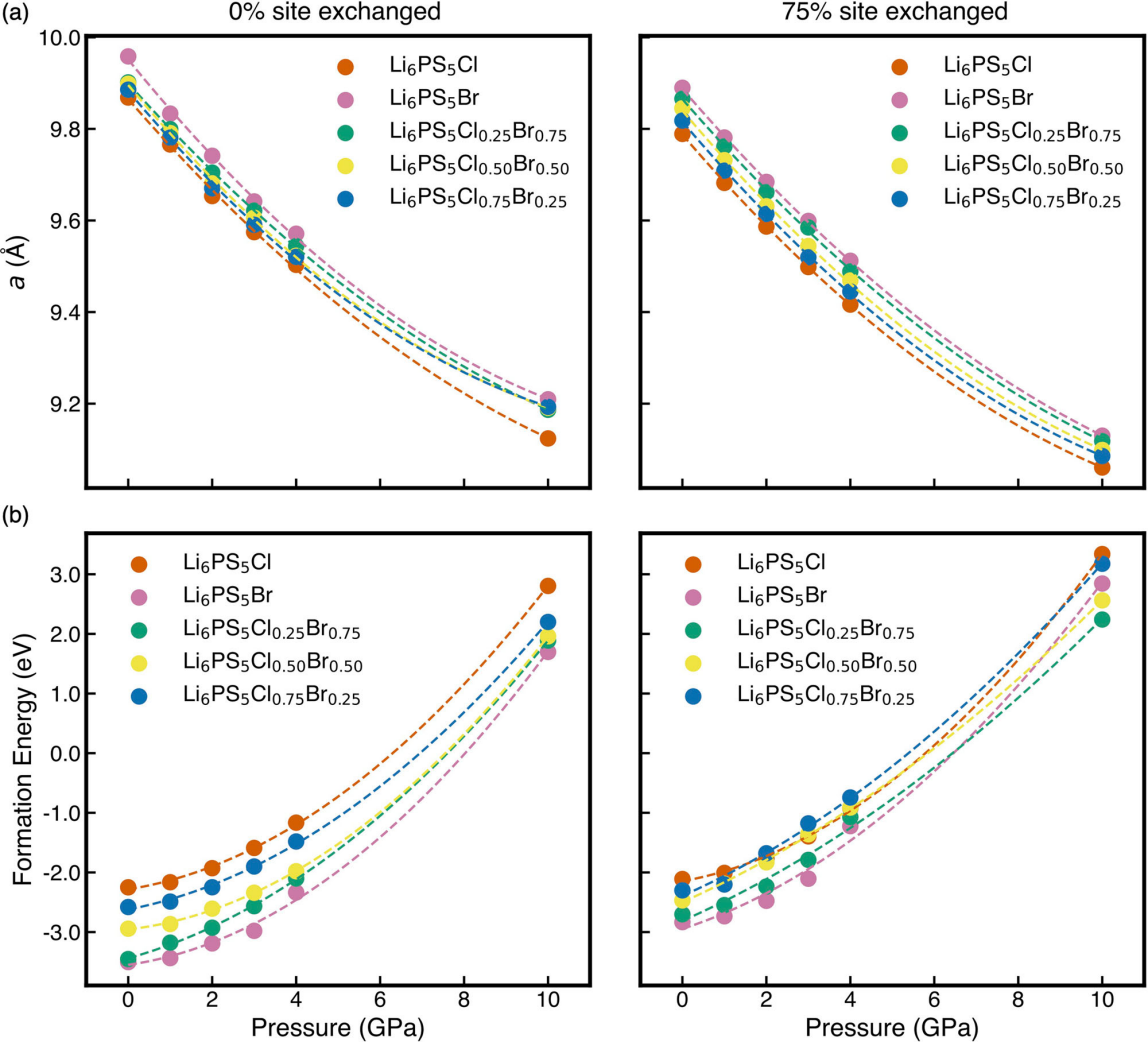
Influence of pressure on structure and formation energy. **a** Unit cell volume and **b** formation energy for Li_6_PS_5_X (X = Cl and/or Br) with 0% and 75% S^2−^/X^−^ site exchange as a function of pressure. The formation energies were calculated with respective to the relevant binary precursors, i.e., LiX (X = Cl or Br), Li_2_S and P_2_S_5_.

We first examined the structural response of Li_6_PS_5_Cl, Li_6_PS_5_Br, Li_6_PS_5_Cl_0.25_Br_0.75_, Li_6_PS_5_Cl_0.50_Br_0.50_ and Li_6_PS_5_Cl_0.75_Br_0.25_ to external pressure for both the 0% and 75% site exchanged structures, as shown in Fig. 2a. Across all pressures, the lattice parameters follow a clear compositional trend governed by the ionic radius of the halide anion. As Br^−^ (1.96 Å) is larger than Cl^−^ (1.81 Å)^45^, Li_6_PS_5_Br exhibits the largest lattice parameter at all pressures, while Li_6_PS_5_Cl shows the smallest. The mixed halide compositions lie between these two endmembers, with their lattice parameters scaling smoothly with Br/Cl ratio. As previously reported for Li_6_PS_5_Br, increasing anion disorder results in reduced unit cell volumes/lattice parameters as a result of the more homogeneous charge distribution of Li ions, leading to contraction of the unit cell due to electrostatic interactions^44^.

Examination of the pressure-dependent lattice parameters of Li_6_PS_5_X (X = Cl and/or Br) reveals a clear contraction with increasing pressure, in agreement with the in-situ X-ray diffraction data of Faka et al. for Li_6_PS_5_Br, who observed reversible compression without phase transitions up to 10 GPa^43^. For the fully ordered structures, the cubic lattice parameters decrease from 9.87 Å (X = Cl), 9.96 Å (X = Br) and 9.90 Å (X = Cl_0.5_Br_0.5_) at 0 GPa to 9.12 Å (X = Cl), 9.21 Å (X = Br), and 9.19 Å (X = Cl_0.5_Br_0.5_) at 10 GPa. The partially disordered (75% site exchanged) structures show a comparable continuous contraction, with lattice parameters decreasing from 9.80 Å (X = Cl), 9.89 Å (X = Br) and 9.84 Å (X = Cl_0.5_Br_0.5_) at 0 GPa to 9.06 Å (X = Cl), 9.13 Å (X = Br) and 9.10 Å (X = Cl_0.5_Br_0.5_) at 10 GPa. The magnitudes of these pressure-induced reductions are similar regardless of the halide nature, which reflects the similar bulk moduli of 28.7 and 29.0 GPa for Li_6_PS_5_Cl and Li_6_PS_5_Br, respectively, calculated from DFT simulations^22^. Such reductions in lattice parameters are expected to have a significant impact on the migration volume for Li ions and therefore the overall Li-ion conductivity^44^.

Figure 2b presents the calculated formation energies of Li_6_PS_5_X (X = Cl and/or Br) from their respective binary precursors (i.e., LiX (X = Cl or Br), Li_2_S and P_2_S_5_) as a function of pressure for both ordered (0%) and partially disordered (75%) structures. All compositions exhibit negative formation energies below 5 GPa, indicating reasonable stability under moderate compression. Although the formation energy of Li_6_PS_5_Br is more exothermic at 0 GPa, it increases slightly more rapidly with pressure compared to the Cl-rich argyrodites, i.e., an average increase 0.520 eV/GPa for Br compared to 0.505 eV/GPa for Cl in the fully ordered structures. This trend becomes more pronounced in the disordered systems (75%), where the rates rise to 0.568 eV/GPa for Br and 0.545 eV/GPa for Cl. These results show that the Cl-rich systems exhibit a smaller pressure-induced destabilisation compared to the Br-rich compositions, consistent with their stiffer bonding environment, stronger electrostatic interactions and lower polarisability^34,46^.

**Fig. 3:**
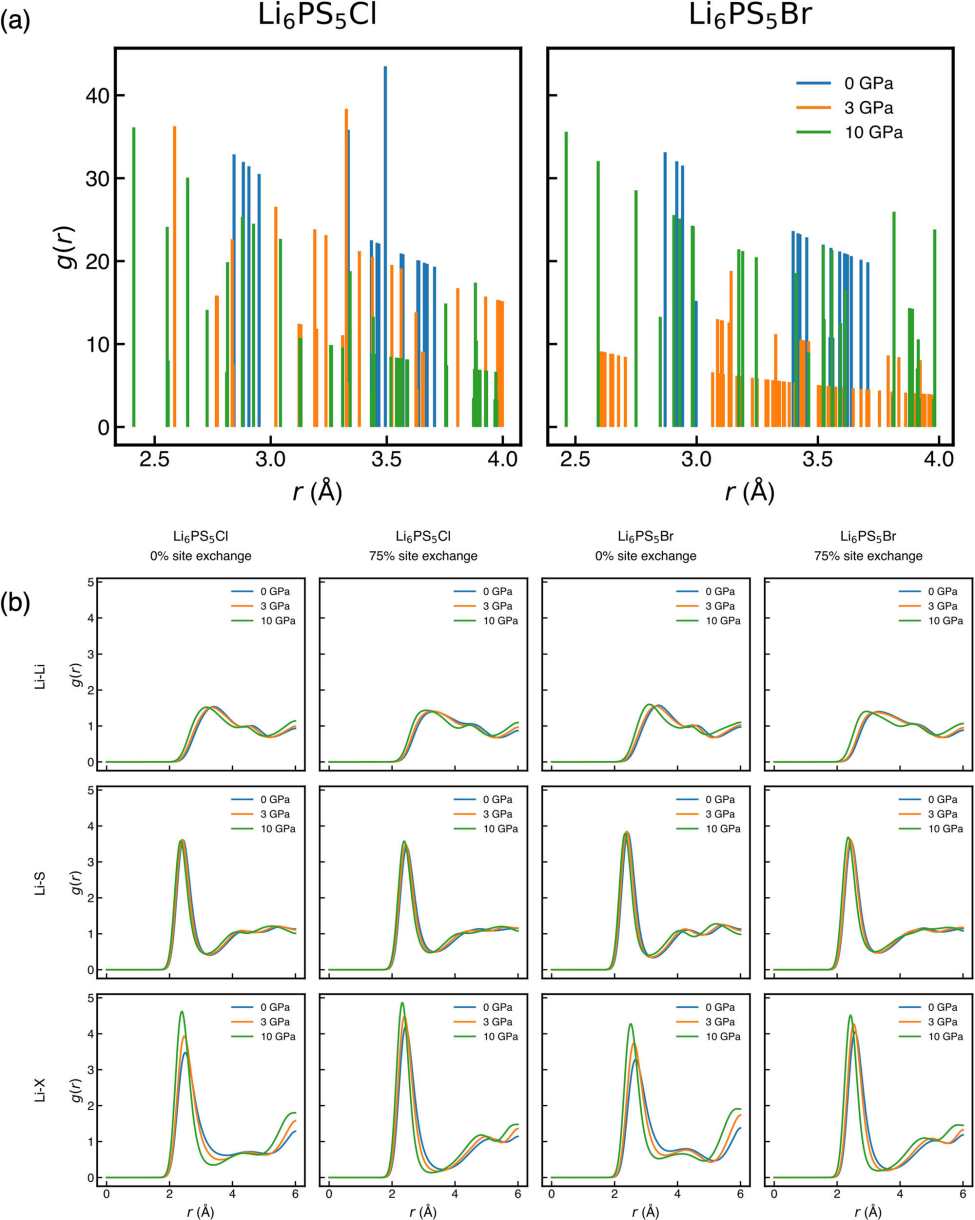
Influence of pressure on local structure. Radial distribution functions, *g*(*r*), for **a** Li–Li in Li_6_PS_5_X (X = Cl or Br) with 0% S^2−^/X^−^ site exchange at 0 K and **b** Li–Li, Li–S and Li–X in Li_6_PS_5_X with 0% and 75% S^2−^/X^−^ site exchange at 600 K under pressures of 0, 3 and 10 GPa. Data for **b** are time averaged from production AIMD trajectories.

Radial distribution functions (RDFs) are plotted in Fig. 3 to provide complementary local structural insights into the effect of pressure on Li_6_PS_5_X (X = Cl and/or Br). Figure 3a shows the Li–Li RDFs for the optimised Li_6_PS_5_X structures with 0% S^2−^/X^−^ site exchange at 0 K. As expected, increasing the external pressure results in a range of shorter Li–Li separations of < 2.8 Å; however, several longer Li–Li distances of > 3.7 Å are also created. The shortest Li–Li separations are associated with Li ions within the same cage (48*h*–24*g*–48 *h* and 48*h*–48 *h* intracage hops, see Fig. 1b), while the longest Li–Li distances arise from Li ions between neighbouring cages (48*h*–48 *h* intercage hops). This is important because it is known that intercage Li-ion hopping represents the rate-limiting process for long-range Li-ion diffusion in these argyrodites^20^. Therefore, as these distances increase, Li-ion diffusion is likely to be restricted with increasing pressure. Furthermore, the reduced Li–Li distances within cages will result in increased Coulombic repulsion, which is likely to also impact the overall conductivity. We also carried out the same analysis for the optimised Li_6_PS_5_X structures with 75% S^2−^/X^−^ site exchange; however, the greater level of distortion and larger range of Li–Li distances made it difficult to draw the same clear conclusion.

We also calculated a range of RDFs from our AIMD simulations at 600 K, as shown in Fig. 3b. At ambient pressure (0 GPa), our calculated RDFs for both the 0% and 75% site exchanged systems are in excellent agreement with previous AIMD studies^28^. For the Li–Li RDFs, a broad distribution of Li–Li separations is observed above 3 Å. The primary peaks in the Li–Li RDFs are smaller and broader in anion site exchanged systems, indicative of a greater range of site–site separations and a more disordered lithium sublattice. In contrast, the Li–anion RDFs present single sharp primary peaks at 2.2–2.9 Å depending on the halide identity and level of site disorder.

In agreement with the RDFs at 0 K, with increasing pressure, the major peaks in each RDF shift to shorter distances; however, the magnitudes of these shifts differ considerably depending on the element pair and composition. For the Li–Li pair, the RDFs for each system at 0 and 3 GPa are reasonably commensurate. In contrast, at 10 GPa, there is a clear change with a substantial reduction in the Li–Li separation distance of the main peak. This reduction is also more significant for Li_6_PS_5_Br compared to Li_6_PS_5_Cl. This change at 10 GPa reflects both the significant lattice compression and more compact Li sublattice. We also observe a higher proportion of Li–Li distances above 5 Å in the systems at 10 GPa, as found for the optimised structures at 0 K. Li–Li separation is well known to have a strong influence on fast Li-ion transport in Li-ion conductors^47,48^. In previous molecular dynamics simulations of γ- and β-Li_3_PS_4_, similar reductions in the Li–Li separation of the main RDF peaks were found to directly contribute to inferior Li-ion transport, in agreement with our current findings for Li_6_PS_5_X at 10 GPa.

A similar phenomenon is also observed for the Li–X RDFs, where there is a significant shift in the peaks to shorter distances with increasing pressure, particularly for the 0% site exchanged systems. At 10 GPa, this shift results in the distance of the main Li–X peak becoming similar or even shorter than for the main Li–S peaks. Such behaviour is also typically observed with a reduction in temperature and the resulting increase in local ordering and decrease in Li-ion conductivity. The Li–X RDFs allow for direct comparison between chloride and bromide coordination environments. Quantitatively, the first peak positions confirm that Li–Br distances are consistently longer than Li–Cl under ambient conditions (2.67 Å for Br and 2.49 Å for Cl at 0 GPa). Under maximum compression (10 GPa), both coordination shells contract, but the Li–Br environment exhibits a larger magnitude of contraction (2.67–2.43 Å, Δ = –0.24 Å) compared to Li–Cl (2.49–2.34 Å, Δ = –0.15 Å), reflecting a softer and more polarisable Br^–^ coordination shell. This softer environment compresses more readily, illustrative of a larger response of Br-rich systems to applied pressure. Interestingly, and as discussed above, this increased rate of contraction with pressure for Li–Br compared to Li–Cl distance is not reflected in the calculated lattice parameters in Fig. 2a. This suggests that the influence of pressure is not uniform for both average and local structure in lithium argyrodites. In contrast to Li–Li and Li–X, the effect of pressure on Li–S distances is more subtle, with only a minor shift to shorter distances, even at 10 GPa.

Overall, the above analysis demonstrates that halide chemistry and anion disorder jointly control the pressure stability of Li_6_PS_5_X. Cl-rich compositions are more rigid and cohesive, whereas Br-rich systems are softer and more responsive to external pressure. These distinctions underpin the pressure-dependent electronic and Li-ion transport behaviour discussed in the following sections.

### Electronic structure

Even though electronic structure is directly connected to electrochemical stability, electronic conduction and resistance to dendrite formation, the influence of external pressure on the electronic structure and bandgaps of solid electrolytes for solid-state batteries has rarely been considered in literature. To understand how pressure affects the electronic structures of Li_6_PS_5_X (X = Cl or Br), we calculated the projected electronic density of states (DOS) at the 0% and 75% site exchanged systems as function of pressure, as shown in Figs. S1 and 4, respectively. The DOS plots for both Li_6_PS_5_Cl and Li_6_PS_5_Br reveal that the valence band maximum is primarily composed of S 3p orbitals, while the conduction band minimum is dominated by P 3 s and 3p states across the entire pressure range^49^.

**Fig. 4:**
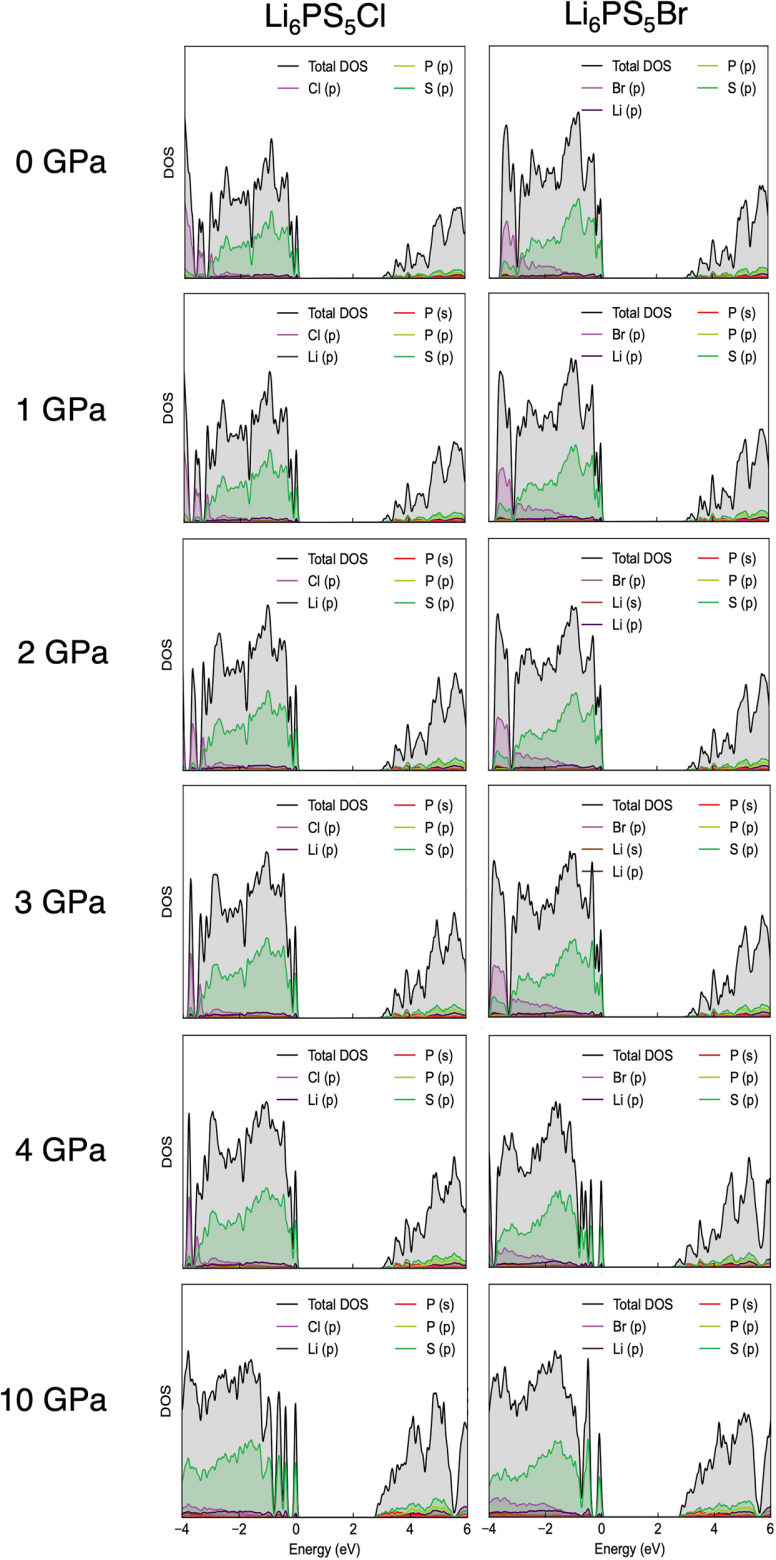
Influence of pressure on electronic structure. Projected electronic density of states (DOS) for Li_6_PS_5_X (X = Cl or Br) with 75% S^2−^/X^−^ site exchange as a function of pressure. Rows show the DOS at increasing pressures of 0, 1, 2, 3, 4 and 10 GPa (from top to bottom). The valence band maximum is set to 0 eV.

In both materials, increasing pressure leads to a broadening of DOS features, reflecting enhanced orbital overlap as the lattice compresses. This is accompanied by a gradual reduction in the bandgap, indicating a tendency toward potentially increased electronic conductivity at high pressure. Li_6_PS_5_Br displays faster smearing of states and more rapid gap narrowing, indicating greater electronic delocalisation and stronger sensitivity to compression, while Li_6_PS_5_Cl retains relatively more defined structure and a slower gap reduction. At low pressure, the valence band is composed of relatively well-defined, closely spaced peaks, primarily arising from S 3p states with some hybridisation from P and the halide. As shown in Fig. 4, with increasing pressure, splitting of the valence band states occurs for the 0% and 75% site exchanged systems due to local structural asymmetry effects in the argyrodite framework, which lift the degeneracy of these states. At 10 GPa, this effect results in isolated bands at the valence band maximum. Interestingly, this behaviour is not observed for the 0% site exchanged systems (Fig. S1), indicating that it is a structural disorder-driven process.

**Fig. 5:**
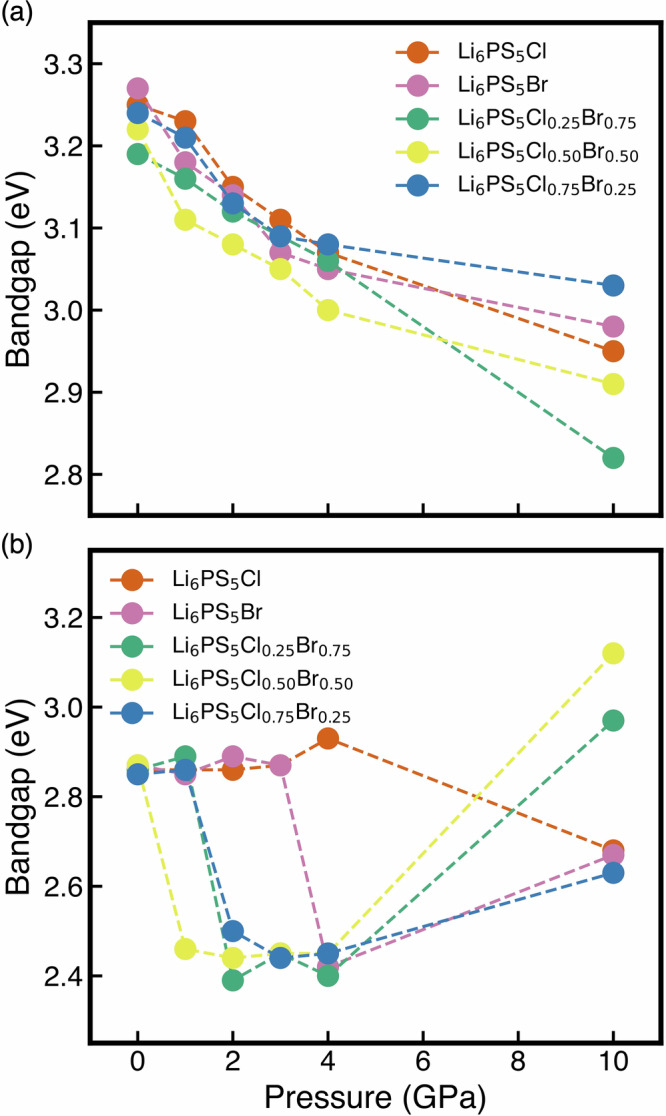
Influence of pressure on bandgap. Bandgaps for Li_6_PS_5_X (X = Cl and/or Br with **a** 0% and **b** 75% S^2−^/X^−^ site exchange as a function of pressure.

The calculated bandgaps for Li_6_PS_5_X (X = Cl and/or Br) with 0% and 75% site exchange as a function of pressure are presented in Fig. 5. For the anion site ordered compositions (0% site exchanged), the bandgaps occupy a narrow range between 3.19 eV (Li_6_PS_5_Cl_0.25_Br_0.75_) and 3.27 eV (Li_6_PS_5_Br) at 0 GPa, in agreement with previous DFT studies^49,50^. For the anion site disordered compositions (75% site exchanged), the bandgaps are remarkably similar (2.85–2.87 eV) at 0 GPa but somewhat smaller than the values obtained for the ordered compositions (3.19–3.27 eV). Given that bandgaps can be used as approximate upper limits of the electrochemical stability window, it is noteworthy that anion site disordering may reduce the stability of these argyrodite solid electrolytes^46,51^. These values show that the halide composition in these argyrodites has minimal impact on their bandgaps and therefore their estimated electrochemical stability window^52^.

As shown in Fig. 5a, with increasing pressure, the bandgaps of anion site ordered Li_6_PS_5_X (X = Cl and/or Br) show a clear decreasing trend. Nevertheless, these decreases are relatively modest ( ~ 0.2–0.5 eV) and are therefore not expected to substantially impact the thermodynamic stability or electronic conductivity of the materials. In contrast, the disordered compositions do not show a clear single trend with increasing external pressure (Fig. 5b). For the Br-containing systems, a notable reduction in bandgap of ~0.4 eV occurs at pressures of 4 GPa. The pressure at which this reduction occurs is different depending on the Br content. Furthermore, it is important to bear in mind that the electronic structure will depend on the particular cation and anion ordering in each composition, which, to some extent, increases variability in the calculated band gaps. This aligns with the experimental findings of Minafra et al.^32^, which show that anion disorder broadens Li^+^ distributions and introduces charge inhomogeneity. At the maximum calculated pressure of 10 GPa, the bandgaps of the Br-containing systems increase by 0.2–0.7 eV, possibly suggestive of a stabilising effect at high pressures. In the case of Li_6_PS_5_Cl, the bandgap remains relatively constant as a function of pressure, with only a small ~0.2 eV at 10 GPa. This result highlights the excellent performance of Li_6_PS_5_Cl as a solid electrolyte for practical solid-state batteries.

The narrowing of band gaps is an important consideration in the development of solid electrolytes as these fast ionic conductors must also have minimal electronic conductivity, i.e., wide band gaps. Whilst the bandgaps calculated here are still too large to any significant levels of electronic conduction, this may not be the case for the grain boundaries of argyrodites, especially when under external pressure. It has been shown that grain boundaries in solid electrolytes can lead to reductions in the bandgap, sometimes with the appearance of highly localised trap states for electrons or holes on ions which are coordinated differently than in the bulk^53^. This is an important consideration for solid electrolyte design as the combined effect of pressure and grain boundaries may lead to significantly narrowed bandgaps with the potential for detrimental electronic conductivity and dendrite initiation^53^.

### Li-Ion transport

**Fig. 6:**
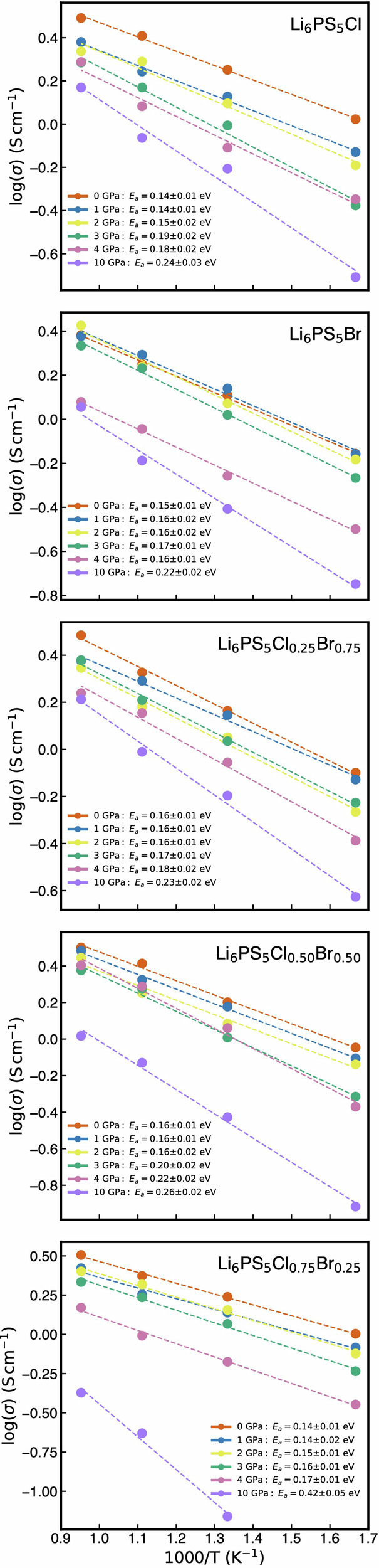
Influence of pressure on Li-ion conductivity. Arrhenius plots of Li-ion conductivity for Li_6_PS_5_X with 75% S^2−^/X^−^ site exchange as a function of pressure.

To elucidate the effects of pressure, halide chemistry and anion disorder on Li-ion transport, temperature-dependent Li-ion conductivities were derived from the AIMD simulations. Figure 6 presents Arrhenius plots of the Li-ion conductivity for Li_6_PS_5_X (X = Cl and/or Br) with 75% S^2−^/X^−^ site exchange under pressures of 0–10 GPa. At ambient pressure (0 GPa), all compositions exhibit high Li-ion conductivities (0.54–1.05 S cm^−1^ at 600 K) and low activation energies in the range of 0.14–0.16 eV, consistent with the superionic behaviour observed experimentally for argyrodites^18,32,35,54,55^. Among the halides, Li_6_PS_5_Cl shows the lowest *E*_*a*_ (0.14 eV), marginally below that of Li_6_PS_5_Br (~0.15 eV), reflecting the more compact Cl-framework that facilitates Li^+^ hopping through interconnected 48 *h* sites via transient occupation of 24 *g* positions^16,19,20,32^. Upon compression, however, conductivity decreases monotonically across all systems, accompanied by a corresponding increase in *E*_*a*_ to 0.22–0.26 eV at 10 GPa. The steeper rise of *E*_*a*_ in Li_6_PS_5_Cl relative to Li_6_PS_5_Br indicates that Li^+^ migration in Cl-rich compositions is more sensitive to lattice densification. Bromide substitution mitigates this effect through enhanced lattice softness, which allows local structural relaxation and partial preservation of the percolating Li^+^ conduction network under pressure^34,56^.

The mixed-halide systems display intermediate behaviour, following the same qualitative trend of conductivity suppression and barrier growth with increasing pressure. However, compositions containing higher Br fractions (Li_6_PS_5_Cl_0.5_Br_0.5_ and Li_6_PS_5_Cl_0.25_Br_0.75_) exhibit the highest pressure resilience, suggesting a synergistic balance between the strong electrostatic framework of Cl^−^ and the softer, more compliant bonding introduced by Br^−^. This trend mirrors the mechanical compliance discussed earlier, where Br-rich frameworks accommodated strain more effectively than Cl-rich analogues.

**Fig. 7:**
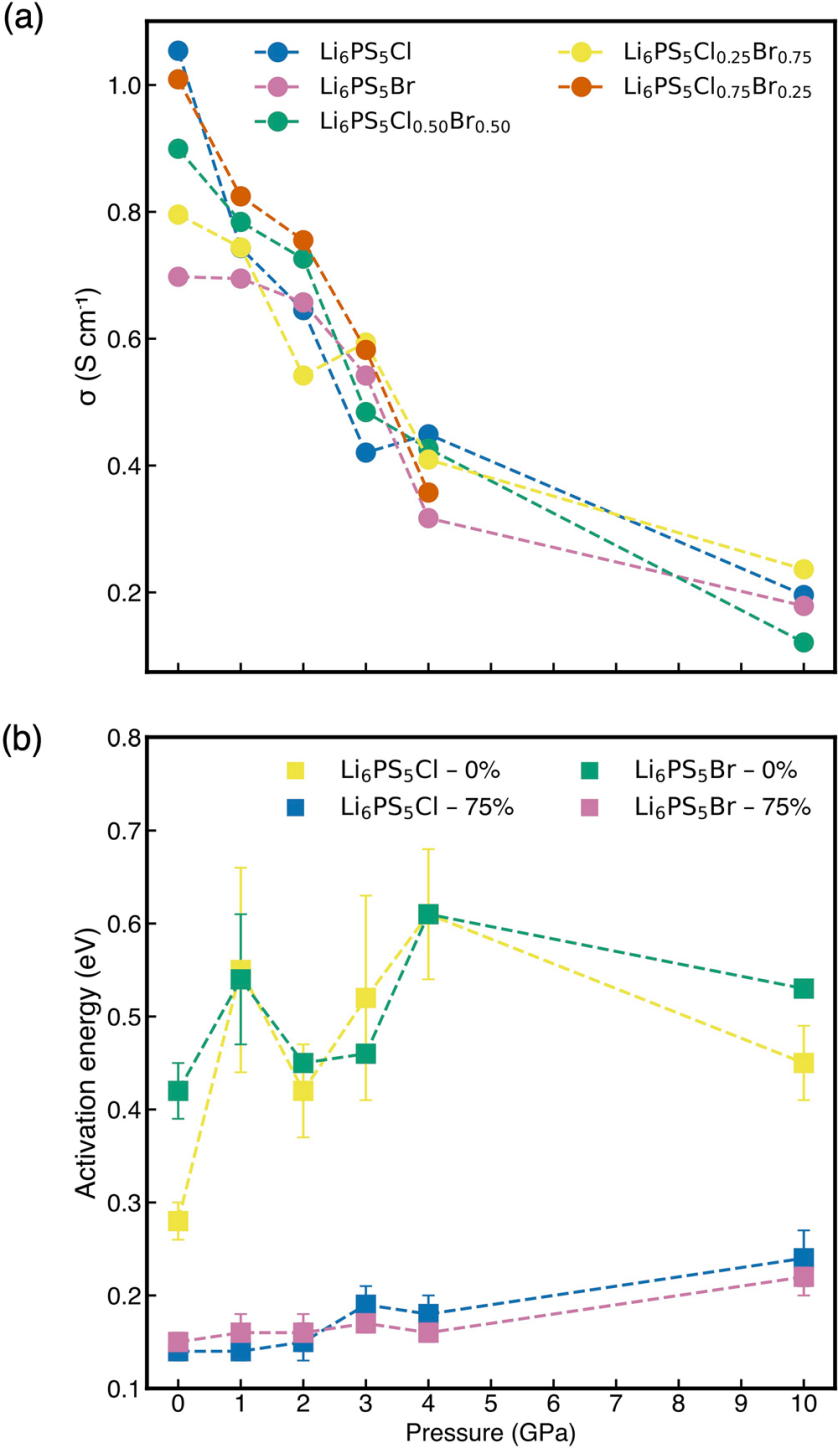
Influence of pressure on Li-ion transport. **a** Pressure dependence of Li-ion conductivity at 600 K for Li_6_PS_5_X (X = Cl and/or Br) with 75% S^2−^/X^−^ site exchange. Insufficient Li-ion diffusion events were observed for the 0% S^2−^/X^−^ site exchanged systems at 600 K so the corresponding conductivities could not be calculated. **b** Pressure dependence of Li-ion activation energy (*E*_*a*_) for Li_6_PS_5_X (X = Cl or Br) with 0% and 75% S^2−^/X^−^ site exchange. The equivalent *E*_*a*_ values for Li_6_PS_5_Cl_0.5_Br_0.5_, Li_6_PS_5_Cl_0.75_Br_0.25_ and Li_6_PS_5_Cl_0.25_Br_0.75_ are given in Fig. S2.

Figure 7 summarises the pressure dependence of conductivity (σ) and activation energy (*E*_*a*_) for Li_6_PS_5_X (X = Cl and Br) with 0% and 75% site exchange. For both Li_6_PS_5_Cl and Li_6_PS_5_Br, introducing 75% S^2−^/X^−^ site exchange significantly lowers *E*_*a*_ relative to the fully ordered (0%) structures, resulting in higher conductivities over the entire pressure range. This behaviour reflects the established role of anion disorder in broadening the distribution of accessible Li sites and enabling more three-dimensional, energetically favourable migration pathways^24,28,30,32,44^.

At the atomistic level, Li-ion diffusion in argyrodites proceeds through hops between 48 *h* cages via transient occupations of 24 *g* sites, forming a three-dimensional percolation network^16,20,24,27,28^. Anion disorder (75% S^2−^/X^−^ site exchanged) enhances this connectivity by perturbing local charge distributions and creating multiple low-energy pathways, while applied pressure acts oppositely by narrowing the bottlenecks between cages. The overall transport response therefore arises from a balance between disorder-induced pathway diversification and pressure-driven lattice densification.

**Fig. 8:**
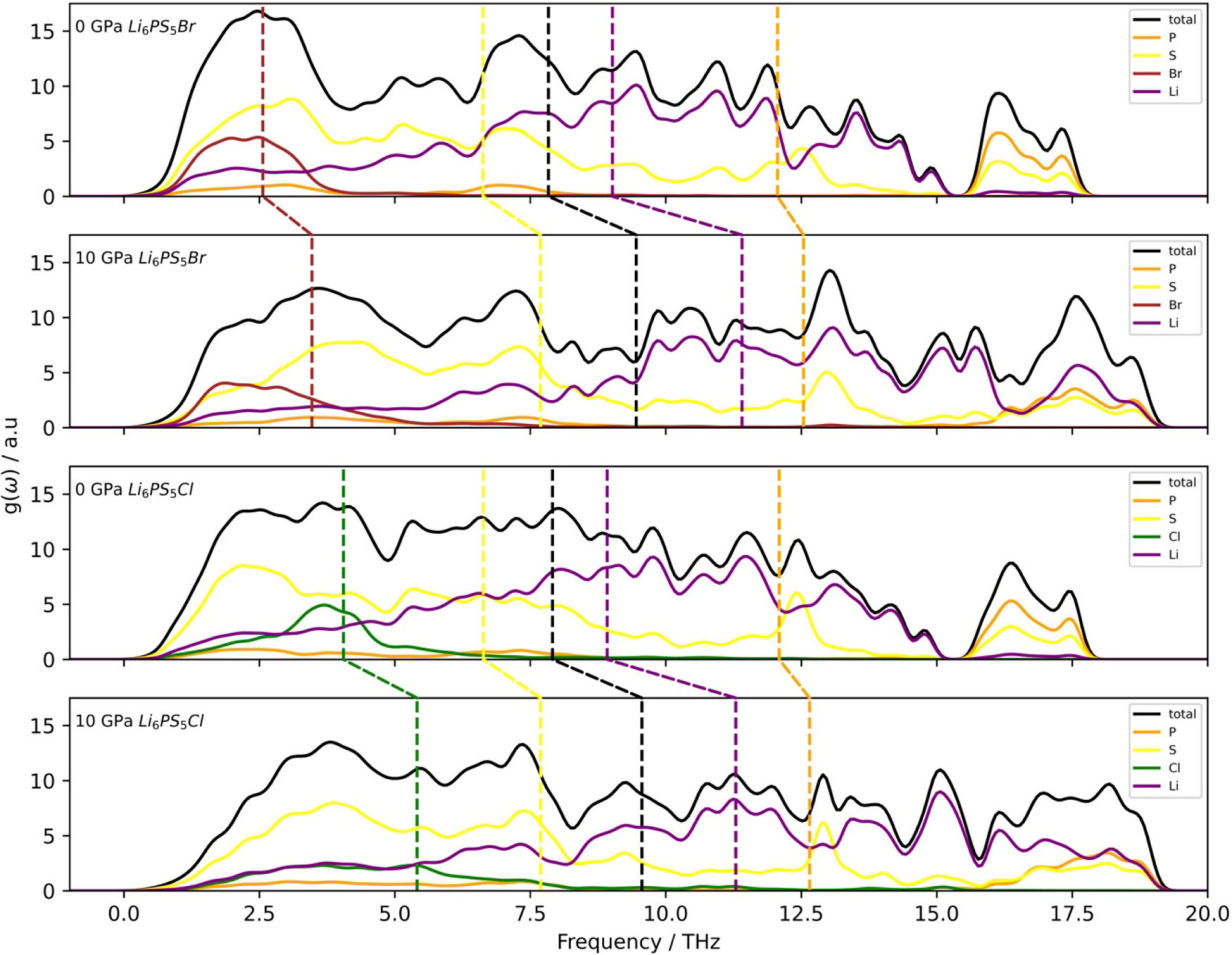
Influence of pressure on lattice dynamics. Partial vibrational density of states (g(ω)) for Li_6_PS_5_X (X = Cl or Br) with 0% S^2−^/X^−^ site exchange at 0 and 10 GPa. PBC values are denoted by dashed vertical lines.

The hardening of phonon modes under pressure is a well-known phenomenon linked to the increase in lattice vibration frequencies as a material is compressed, caused by the shortening of atomic bonds and increased interatomic force constants. Exploration of the lattice dynamics properties for Li_6_PS_5_X (X = Cl or Br) at 0 and 10 GPa revealed entirely real vibrational frequencies for each structure, suggesting that the pressurised optimised structures are dynamically stable (phonon dispersion plots are shown in Fig. S3). The application of pressure leads to general vibrational hardening of modes, reflected in both the g(ω) spectra, as well as the total (all ion) and ion projected PBC values. The g(ω) for each material are shown in Fig. 8, with the PBC values shown by dashed vertical lines, which are connected across the two pressure values for each Li_6_PS_5_X composition.

It is clear from the PBC values that there is a general shift upwards in vibrational frequency (hardening) for each ionic species (and therefore also the all-ion PBC), with the most significantly upshifted species being the Li ions for both Li_6_PS_5_X (X = Cl or Br). Vibrational hardening of phonons under pressure is most likely a result of a decrease in interatomic distances leading a stiffer lattice and an increase in restoring force of vibrations, and has been widely reported for a number of different materials and molecular solids^57–59^. The Li projected PBC has been correlated to some success with ion transport (both in terms of the Arrhenius prefactor and activation energy)^34^ for a number of Li-ion conductors, with lower PBC (soft) lattices typically exhibiting increased Li conductivity as the vibrations are more easily excited and assist with ionic migration^60–62^. The increase in Li-ion PBC as a result of the application of pressure for the Li_6_PS_5_X (X = Cl or Br) materials considered in this work is in agreement with the calculated decrease in σ, further consolidating the negative influence of pressure on Li-ion conductivity in argyrodites.

Our results demonstrate that Li-ion transport in Li_6_PS_5_X is highly sensitive to external pressure, with both halide chemistry and anion disorder governing the extent of this response. Cl-rich compositions show lower activation barriers under ambient conditions but a greater loss of conductivity under higher pressures (3 GPa), indicating a stronger sensitivity to densification. In contrast, Br-analogues, particularly those with substantial disorder (75%), exhibit a more resilient transport response, consistent with the greater chemical softness and structural compliance of the Br-substituted lattice. In addition to the negative influence of pressure on Li-ion conductivity observed in this work, it has been previously shown that external pressure can be used to induce dislocations in Li_6_PS_5_Br, which can actually enhance its Li-ion conductivity^43^. These findings therefore emphasise that pressure, halide substitution and anion disorder are interdependent design parameters for optimising argyrodite solid electrolytes capable of sustaining high ionic conductivity under mechanical stress and internal strain.

## Discussion

The understanding of pressure effects in solid electrolytes is essential for their large-scale utilisation in solid-state batteries. Despite this fact, the role of pressure in these materials has been so far underexplored, particularly using atomistic modelling. In this work, we have investigated how pressure influences the structure, electronic properties and Li-ion conductivity in the lithium argyrodite family Li_6_PS_5_X (X = Cl and/or Br) and considered its intricate interplay with halide mixing and S^2−^/X^−^ anion disorder at the atomic scale. The key results are summarised as follows:

1. All Li_6_PS_5_X (X = Cl and/or Br) compositions remain structurally coherent under compression up to 10 GPa, with continuous lattice contraction and no evidence of phase transitions.
2. The impact of pressure on the average and local structure is not uniform. For example, while the magnitude in the reduction of the lattice parameter with pressure is similar for all Li_6_PS_5_X compositions, the primary Li–Br distance decreases more substantially with increasing pressure than the primary Li–Cl distance. Furthermore, whilst the intracage Li–Li distances are shortened with increasing pressure, the distance between neighbouring Li cages can increase.
3. Electronic structure analysis indicates wide bandgaps (2.8–3.3 eV) that are largely insensitive to halide composition. Anion disorder slightly narrows the bandgap and pressure induces modest, composition-dependent variations that are not expected to significantly affect electrochemical stability.
4. Li-ion conductivities remain high (0.54–1.05 S cm^−1^ at 600 K) and activation energies low ( ~ 0.14–0.16 eV) up to ~ GPa, confirming robust Li-ion transport pathways under moderate compression.
5. At higher pressures, Li-ion conductivities decrease and activation energies increase, with Br-rich and partially disordered structures showing the strongest resistance to pressure-induced transport degradation owing to their greater lattice flexibility and polarisability.

From a design perspective, our results demonstrate that the interplay of pressure, halide chemistry and anion disorder critically governs the key properties of argyrodites as solid electrolytes, including Li-ion conductivity and electrochemical stability. These insights help to rationalise experimental observations of pressure-sensitive Li-ion transport in Li_6_PS_5_X and establish practical design rules for engineering solid electrolytes that maintain high performance in solid-state batteries under external pressure constraints.

## Methods

### DFT calculations

DFT calculations were performed using the projector-augmented wave (PAW) method, as implemented in the Vienna *Ab Initio* Simulation Package (VASP)^63,64^. The Perdew-Burke-Ernzerhof for solids (PBEsol) generalised gradient approximation (GGA) was used to describe exchange-correlation effects. Given that the Li ions partially occupy the 48 *h* site in Li_6_PS_5_X, a screening over the possible configurations was performed using pymatgen^65^. After enumerating the possible structures, 50 were selected through minimisation of their electrostatic energies and then fully relaxed using DFT. The configuration with the lowest energy was identified and used for subsequent simulations. A similar process was also applied for the generation of the mixed halide and anion site-disordered structures. All structural optimisations and energy calculations employed a plane-wave cutoff energy of 600 eV and Brillouin zone integrations were performed using a 2 × 2 × 2 *k*-point mesh. Energy and force convergence criteria of 10^−5^ eV and 0.01 eV Å^−1^, respectively, were used throughout. Structural optimisations were completed with the complete relaxation of atomic position, cell shape, and cell volume, under applied pressures of 0, 1, 2, 3, 4, and 10 GPa. Clearly, these pressures are far beyond the scope of typical device operation values but are appropriate for probing intrinsic or limiting behaviour rather than practical regimes. Furthermore, it is noteworthy that pressures as high as 10 GPa have been successfully used to probe pressure effects on argyrodites experimentally^43^. Density of states (DOS) calculations were performed using the HSE06 hybrid functional for a more accurate electronic structure^66^. The sumo Python toolkit was used for postprocessing of the DOS data^67^.

### AIMD simulations

AIMD simulations were performed in the canonical (NVT) ensemble, using the Nose-Hoover thermostat, to analyse a range of optimised structures incorporating varying degrees of anion site disorder and halide mixing as a function of pressure. The plane-wave energy cutoff was reduced to 400 eV and a single *k*-point mesh (Γ-point) was used. Given the substantial number of AIMD simulations required to consider the effects of pressure, halide mixing and anion site disorder ( > 240 combinations), only a single Li_6_PS_5_X unit cell (52 atoms) was used. Although the use of this small cell may lead to an overestimation of absolute conductivities resulting from finite size effects, our focus is on trends rather than absolute values. This system size also enabled us to conduct simulations over a total duration of 200 ps at four temperatures (600–1050 K at 150 K intervals) with a 2 fs timestep. Li-ion diffusion coefficients (*D*_Li_) were obtained from the mean squared displacement (MSD) of Li ions during the simulations:

1 $${\rm{\langle }}{r}_{i}^{2}(t){\rm{\rangle }}=6{D}_{{\rm{Li}}}t$$

where $\langle {r}_{i}^{2}(t)\rangle$ represents the MSD and t is time. The Li-ion diffusion coefficients were subsequently converted into Li-ion conductivities (*σ*) using the Nernst-Einstein equation.

### Phonon Calculations

Harmonic phonon calculations were conducted on the structures of both Li_6_PS_5_X (X = Cl or Br) with no S^2−^/X^−^ site disorder at 0 and 10 GPa to explore the influence of pressure on the vibrational characteristics of the materials. The optimised geometries were further optimised for these calculations to an ionic force convergence of 0.001 eV Å^−1^ with a 2 × 2 × 2 k-point mesh. The lattice dynamics calculations were completed using the finite differences approach, as implemented in PHONOPY^68^. The optimised geometries were expanded to 2 × 2 × 2 supercells, which were sampled on a proportionally smaller k-point mesh (Γ-point). Long range electrostatic interactions were accounted for using the non-analytical term correction, as implemented in PHONOPY, using the static dielectric constant tensor and born effective charges calculated using VASP. The phonon density of states (g(${\rm{\omega }}$)) was calculated on a 10 × 10 × 10 mesh of points. To explore the influence of pressure on the ionic g(ω) spectra (partial vibrational density of states), the phonon band centre (PBC) was calculated using the following equation:

2 $${PBC}=\frac{\int \omega \times g\left(\omega \right).d\omega }{\int g\left(\omega \right).d\omega }$$

where ω represents a vibrational frequency and g(ω) represents either the total (all ion) or ion projected phonon density of states for the all ion or projected PBC, respectively.

## Supplementary information

Supplementary Information

## Data Availability

The datasets generated and/or analysed during the current study are not publicly available as they form part of an ongoing research programme and are subject to further analysis but are available from the corresponding author on reasonable request.
