# Supplementary material for: Influence of pressure on structure, stability and ionic conductivity of lithium argyrodite solid electrolytes
Source: NPJ Energy Mater. 2026 Jul 22;1(1):7. doi: 10.1038/s44456-026-00009-1 (PMC13446071; doi:10.1038/s44456-026-00009-1)
Supplement: Supplementary file 1 [file 44456_2026_9_MOESM1_ESM.pdf]

—Supporting Information—

Influence of Pressure on Structure, Stability and Ionic Conductivity of Lithium Argyrodite Solid Electrolytes

Jemma L. Cox[1], Jack M. Hemingway[1], James A. Quirk[1], Erli Lu[2] and James A. Dawson[1,*]

[1]*Chemistry – School of Natural and Environmental Sciences, Newcastle University, Newcastle upon Tyne, UK*

[2]*School of Chemistry, University of Birmingham, Birmingham, UK*

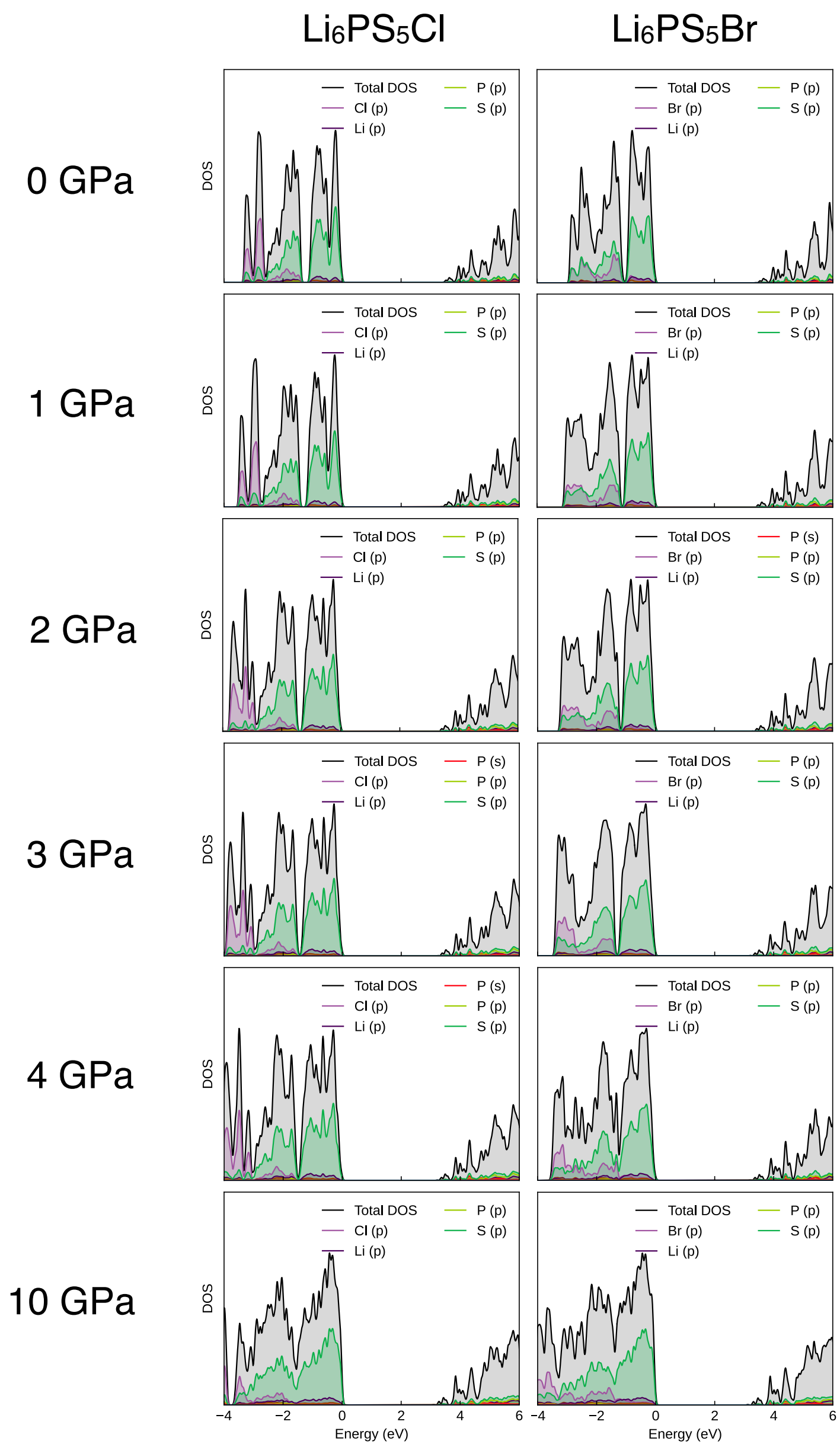

Figure S1. Projected electronic density of states for Li_6PS_5X (X = Cl or Br) with 0% site exchange as a function of pressure. The valence band maximum is set to 0 eV.

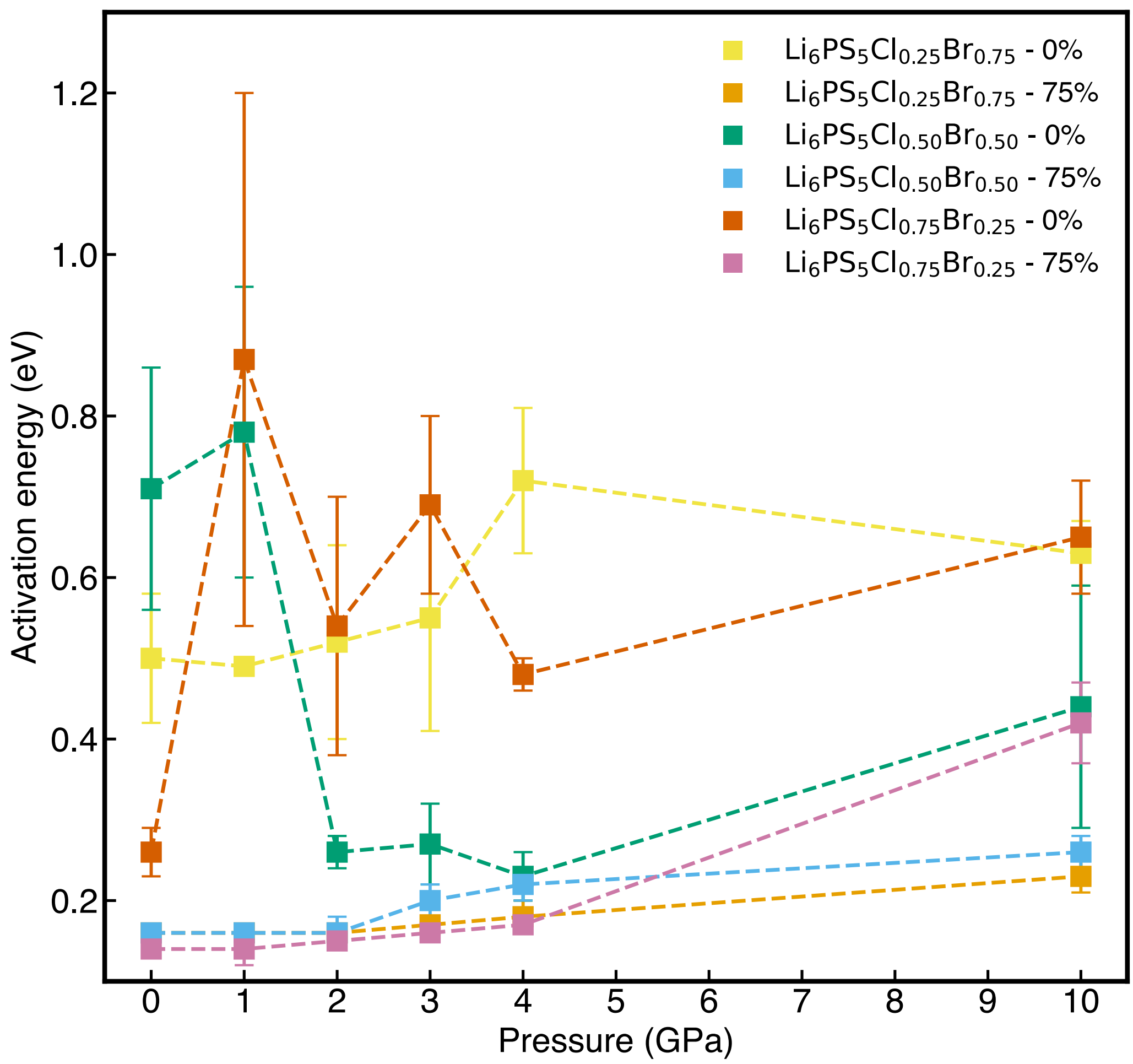

Figure S2. Pressure dependence of Li-ion activation energy (E_a) for $Li_6PS_5Cl_{0.5}Br_{0.5}$, $Li_6PS_5Cl_{0.75}Br_{0.25}$ and $Li_6PS_5Cl_{0.25}Br_{0.75}$ with 0% and 75% S^{2-}/X^- site exchange.

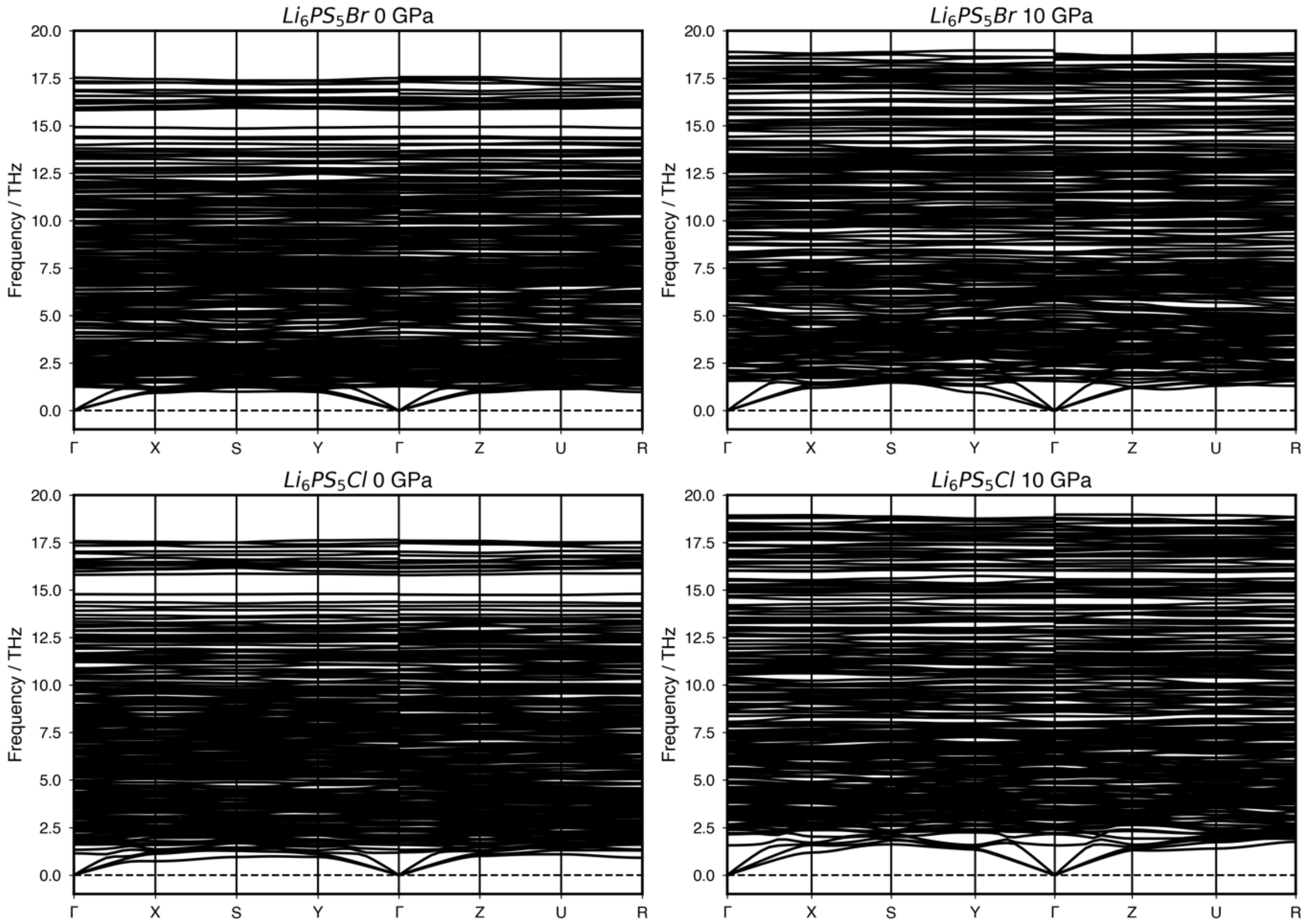

Figure S3. Phonon dispersion spectra of Li_6PS_5X (X = Cl and Br) at 0 and 10 GPa.
